# Dietary valine improved growth, immunity, enzymatic activities and expression of TOR signaling cascade genes in rainbow trout, *Oncorhynchus mykiss* fingerlings

**DOI:** 10.1038/s41598-021-01142-4

**Published:** 2021-11-11

**Authors:** Ishtiyaq Ahmad, Imtiaz Ahmed, Nazir A. Dar

**Affiliations:** 1 Fish Nutrition Research Laboratory, Department of Zoology, University of Kashmir, Hazratbal, Srinagar, 190 006 India; 2 Department of Biochemistry, University of Kashmir, Hazratbal, Srinagar, 190 006 India

**Keywords:** Ichthyology, Biochemistry, Immunology, Physiology, Zoology

## Abstract

This study was conducted to determine the effects of dietary valine (Val) on growth, hemato-biochemical parameters, immunity, enzymatic activities, antioxidant status and expression of target of rapamycin (TOR) and 4E-BP genes in rainbow trout, *Oncorhynchus mykiss* (1.57 ± 0.03 g; 5.10 ± 0.34 cm)*.* Six isonitrogenous (450 g kg^−1^) and isoenergetic (20.90 kJ 100 g^−1^_,_ gross energy) diets were designed to represent varied Val levels (10.5, 13.0, 15.5, 18.0, 20.5 and 23.0 g kg^−1^ dry diet basis). Growth parameters improved significantly (*P* < 0.05) with the amelioration of dietary Val level up to 18.0 g kg^−1^. Highest (*P* < 0.05) body protein content was noted at 18.0 g kg^−1^ dietary Val. Significant differences in hematological, intestinal enzymatic activities and antioxidant parameters were noted. However, plasma variables did not show any significant differences except aspartate transaminase and uric acid. Total protein content increased significantly, while the albumin and globulin content did not show any significant (*P* > 0.05) difference. Moreover expression of TOR mRNA and elF4E-binding protein (4E-BP) was observed higher (*P* < 0.05) at 18.0 g kg^−1^ Val. On the basis of results, optimum dietary Val requirement for maximal growth of rainbow trout was determined to be 18.19 g kg^−1^ of dry diet, corresponding to 40.42 g kg^−1^ of dietary protein.

## Introduction

Protein is yet to be considered as an expensive dietary supplement that supporting the growth performance of fish. However, an understanding of fish protein requirement is of inadequate value devoid of essential amino acids (EAAs) requirement data^1^. Amino acids are considered as vital biological compounds of animals including fish, as they are treated not only as building blocks of proteins, but also participate in various metabolic pathways, nutritional physiology, behavior, sexual metamorphosis of fish etc.^2–4^. Supplement feeding is considered as one of the critical factors influencing growth, well-being and physiology of fish, while the commercial viability of fish culture mainly depends upon marketability and value of production. The major portion of the production cost (40–70%) mainly lies on the fish feed^5–7^. The success of fish farming primarily relies upon the accessibility of satisfactory amount of nutritionally balanced supplemented feeds in the form which is appropriate for the fish^8^. The advancement of nutritionally competent and cost-effective diets for cultured fish species is of immense concern to the commercial success of aquaculture^9,10^. However, the challenges faced in aquaculture are additionally for incorporation of compatible, digestible, less wasteful and cost-effective diets. The development of such diets must subject to meet the nutritional requirements of species specific based balanced feed and its appropriate feeding practices^11,12^.

Valine (Val), isoleucine and leucine represents the family branched chain amino acids (BCAAs). Val is known to have a vital role in several physiological and metabolic reactions and in the growth of various preruminant and monogastric terrestrial animals^9,13^, besides take part in the synthesis of protein and amine neurotransmitters serotonin^14^. In addition to this, Val is also involved in several processes such as protein synthesis, repairement of tissues and nitrogen balance in fish^1,15^. Since, fish cannot synthesize these amino acids including Val, therefore it must be supplied through the diet^16^. The dietary Val requirements for optimum growth have been established for different fish species and are reported in the range from 17.7 to 48.2 g kg^−1^ of dietary protein. Some of the noticeable work which has been reviewed by Zhou et al.^17^ as Lake trout, *Salvelinus namaycush* (17.7–22.3 g kg^−1^)^18,19^, red sea bream, *Pargus major* (20.0 g kg^−1^)^1^, channel catfish, *Ictalurus punctatus* (29.6 g kg^−1^)^20^, red drum, *Sciaenops ocellatus* (32.0–35.0 g kg^−1^)^21^, Indian major carp, *Cirrhinus mrigala* (38.7 g kg^−1^)^9^, blunt snout bream, *Megalobrama amblycephala* (37.1–38.8 g kg^−1^)^22^, rainbow trout, *Oncorhynchus mykiss* (38.5–41.0 g kg^−1^)^13^, Jian carp, *Cyprinus carpio* var. Jian (40.0 g kg^−1^)^23^, Nile tilapia, *Oreochromis niloticus* (41.1–45.3 g kg^−1^)^24^, golden pompano, *Trachinotus ovatus* (46.0–47.0 g kg^−1^)^25^ and grass carp, *Ctenopharyngodon idella* (47.7–48.2 g kg^−1^)^26^. However, it has also been reported that deficiency of Val in the diet can cause weight loss, growth retardation and poor feed conversion ratio, while an excess amount of Val in the diet of fish results in depressive effects^9^.

To determine the metabolic, nutritional and well-being status of fish in response to nutritional enhancements, hematological parameters are considered to be imperative^27–33^. Xiao et al.^24^ found significant variations in some hematological parameters in Nile tilapia fed different valine levels. Other than hematology, nutrient digestibility as well as its utilization can be determined by monitoring the enzymatic profile^34,35^. It has been seen that fluctuations in the concentration of plasma protein, glucose, cholesterol and other action in plasma may be because of explicit markers of sympathetic activation in response to hypoxic stress^36,37^. These parameters are likewise recognized as markers of nutritional status of fish^38,39^. Xiao et al.^24^ reported that total plasma protein concentration was influenced by the dietary valine levels. They also found significant changes in plasma alanine transaminase (ALT) and aspartate transaminase (AST) up to a certain point. Huang et al.^25^ also found some significant variations in serum biochemical parameters in golden pompano, *Trachinotus ovatus* fed varied levels of Val diet. Val is additionally involved in enhancing fish growth by augmenting both digestive as well as absorptive capacity and furthermore influencing intestinal microfloral balance. The intestinal immune response is critically significant for the support of intestinal well-being in fish^40,41^ and is additionally related with its structural integrity^41^. Some previous studies pertaining to intestinal immune responses in fish fed varied concentrations of Val showed significant alterations in some parameters^23,25^, which confirms that appropriate level of dietary Val could improve the fish health.

Branched chain amino acids especially Val performs different immune functions in fish and regulates several key metabolic pathways in response to infectious pathogens^42^. To the best of our knowledge, only few studies are reported about the effects of Val on immunity such as Xiao et al.^24^ reported that optimum supplementation of dietary Val can improve the non-specific immune function of juvenile Nile tilapia. Similar findings were reported for juvenile hybrid grouper, *Epinephelus fuscoguttatus* x *E. lanceolatus* and juvenile red sea bream, *Pargus major* fed with varied concentrations of Val^1,17^. However, some studies revealed that in-deficiency or excess of Val in the diet could resultant in the impairment of immune function, besides increasing susceptibility to disease by disrupting proliferation of liver-associated lymphocytes and cytotoxic T lymphocytes^43,44^. Therefore, appropriate inclusion of Val in the diet could resume defense mechanisms of host i.e., activity of natural killer cells and phagocytic function of neutrophils^45^.

In mammals, target of rapamycin (TOR) signaling pathway has become a main regulator of the inflammatory reaction in monocytes, macrophages and peripheral myeloid dendritic cells^46^. However, in fish, protein synthesis also involves the activation of the TOR signaling pathway^47^. Studies on rainbow trout, *O. mykiss*^48–50^ and Jian carp, *C. carpio* var. Jian^35,51,52^ have reported that nutritional factors could possibly promote protein synthesis by activating the TOR signaling pathway in aquatic animals including fish similar to mammals. It has been reported earlier that Val may activate the mTOR signaling pathway through its downstream effectors S6 kinase (S6K1) and elF4E-binding protein (4E-BP) in bovine mammary epithelial cells^53^, while more recently Zhou et al.^17^ also observed that optimal dietary Val in fish is capable to promote protein synthesis via TOR/S6K1 signaling pathway. However, to the best of our knowledge, only one or two studies are reported on the dietary effects of Val on the expression of TOR signaling pathway in fish, hence needs further re-validation.

In India, rainbow trout is considered as one of the excellent cultured fish species, and its optimum dietary levels of several nutrients including protein requirement (450.0 g kg^−1^ of dietary protein)^54^, leucine (38.77 g kg^−1^ of dietary protein)^55^ and isoleucine (29.95 g kg^−1^ of dietary protein, under publication) have been established in our previous studies. However, no such data are presently available on the dietary Val requirement of rainbow trout by using current advanced technology and techniques, therefore, the present study aimed to explore the impact of dietary Val levels on growth, hemato-biochemical parameters, non-specific immune response, intestinal enzymatic activities, antioxidant properties and expression of TOR and 4E-BP genes in rainbow trout fingerlings.

## Results

### Growth performance

**Figure 1:**
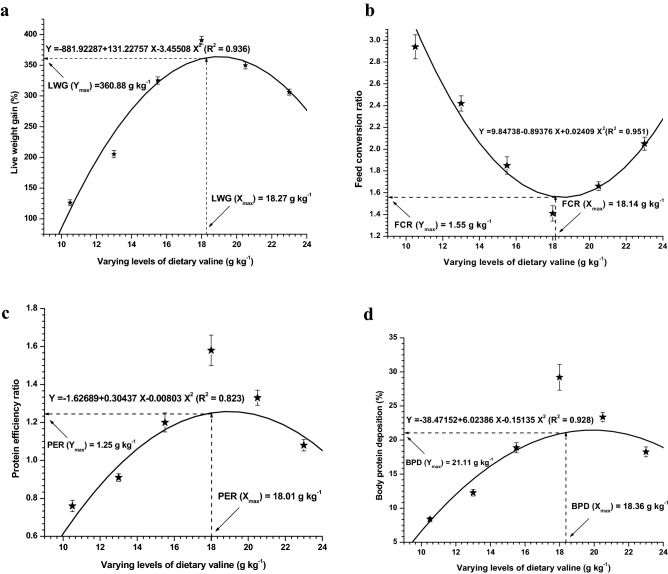
Second-degree polynomial relationship between (**a**) live weight gain (LWG%), (**b**) feed conversion ratio (FCR), (**c**) protein efficiency ratio (PER) and (**d**) body protein deposition (BPD) to dietary valine levels of *Oncorhynchus mykiss* (g kg^−1^).

Growth data generated after the end of 8-week feeding experiment are presented in Table 1. Survival was not affected with respect to each dietary treatment, except the lowest diet i.e. 10.5 g kg^−1^ Val diet, where 98% survival was recorded. The live weight gain (LWG%) obtained with respect to each diet showed significant (*P* < 0.05) improvement by increasing the concentrations of dietary Val and reported a plateau at diet 18.0 g kg^−1^, represented highest LWG%, afterwards it showed slightly decreasing trend. Specific growth rate (SGR), protein efficiency ratio (PER) and body protein deposition (BPD) showed similar pattern to that of LWG%. However, fish fed 18.0 g kg^−1^ supported best feed conversion ratio (FCR) followed by 16.0 g kg^−1^ Val supplemented diet. Quadratic regression analysis of LWG%, FCR, PER and BPD against dietary Val levels indicated that optimal dietary Val levels for rainbow trout was 18.27, 18.14, 18.01 and 18.36 g kg^−1^ dietary Val, respectively (Fig. 1a,b,c,d). The hepatosomatic index (HSI) values showed significant (*P* > 0.05) differences with respect to different Val containing diets, with highest HSI content was noted at starting diets i.e. 10.5 and 13.0 g kg^−1^.

**Table 1:** Growth, FCR, protein deposition and percentage survival of rainbow trout, *Oncorhynchus mykiss* fingerlings fed diets containing varying levels of dietary valine for 8-weeks (mean values of 3 replicates + SEM; *n* = 3)^*^

	Dietary val levels (g kg^−1^)						
	10.5	13.0	15.5	18.0	20.5	23.0	P-value
Average initial weight (g)	1.589 ± 0.02	1.540 ± 0.04	1.527 ± 0.03	1.510 ± 0.05	1.563 ± 0.02	1.583 ± 0.04	0.14029
Average final weight (g)	3.597 ± 0.12^f^	4.708 ± 0.23^e^	4.907 ± 0.21^d^	7.416 ± 0.32^a^	7.032 ± 0.16^b^	6.435 ± 0.20^c^	0.13818
Live weight gain (%)^a^	126.190 ± 4.77^f^	205.33 ± 5.97^e^	325.170 ± 6.0^c^	390.63 ± 6.55^a^	349.76 ± 5.69^b^	306.23 ± 4.90^d^	0.04441
Specific growth rate^b^	1.46 ± 0.04^e^	1.99 ± 0.03^d^	2.58 ± 0.02^c^	2.84 ± 0.02^a^	2.68 ± 0.02^b^	2.50 ± 0.06^d^	0.02490
Feed conversion ratio^c^	2.94 ± 0.11^a^	2.42 ± 0.07^b^	1.85 ± 0.08^d^	1.41 ± 0.07^e^	1.66 ± 0.04^f^	2.05 ± 0.06^c^	0.02716
Protein efficiency ratio^d^	0.76 ± 0.03^e^	0.91 ± 0.02^d^	1.20 ± 0.05^c^	1.58 ± 0.08^a^	1.33 ± 0.04^b^	1.08 ± 0.03^d^	0.01085
Body protein deposition (%)^e^	8.39 ± 0.25^e^	12.27 ± 0.48^d^	18.88 ± 0.74^c^	29.21 ± 1.89^a^	23.36 ± 0.68^b^	18.26 ± 0.73^c^	0.01724
HSI^f^	3.07 ± 0.15^a^	2.83 ± 0.10^b^	2.51 ± 0.12^c^	2.58 ± 0.10^c^	2.43 ± 0.12^d^	2.40 ± 0.15^d^	0.03929
Survival (%)	93	100	100	100	100	100	0.07057

^a^Weight gain (%), Final body weight–initial body weight/initial weight × 100.

^b^Specific growth rate (SGR %) = 100 × (In final wet weight (g)-In initial wet weight g)/duration (days).

^c^Feed conversion ratio (FCR) = Dry weight of feed consumed / Wet weight gain.

^d^Protein efficiency ratio (PER) = Wet weight gain (g) / Protein consumed (g, dry weight basis).

^e^Body protein deposition (BPD %) = 100 × (BWf × BCPf) – (BWi × BCPi) / [TF × CP].

Where BWi and BWf = mean initial and final body weight (g), BCPi and BCFf = mean initial and final percentage of muscle protein,

TF = Total amount of diet consumed and CP = Percentage of crude protein of the diet.

^f^HSI, Hepatosomatic index.

LWG_y_ = – 881.92287 + 131.22757 X – 3.45508 X2 (R^2^ = 0.936).

FCR_y_ = 9.84738 – 0.89376 X + 0.02409 X2 (R^2^ = 0.951).

PER_y_ = –1.62689 + 0.30437 X – 0.00803 X2 (R^2^ = 0.823).

BPD_y_ = –38.47152 + 6.02386 X – 0.15135 X2 (R^2^ = 0.928).

### Whole body composition

Incremental level of dietary Val had affected whole body composition of rainbow trout, *O. mykiss* fingerlings and the data are presented in Table 2. Moisture content showed linearly decreased pattern with increasing concentrations of each dietary Val levels with lowest moisture content 72% was recorded with fish fed 23.0 g kg^−1^ Val containing diet. The body protein content significantly (*P* < 0.05) increase with the elevated concentrations of each dietary Val level up to 18.0 g kg^−1^, and thereafter reduction in protein content was noted. Whole body fat content showed an exponential pattern with each incremental Val levels and produced maximum fat content at 23.0 g kg^−1^ Val fed diet. While, higher body ash content was noted in fish receiving lower dietary doses of Val containing diets i.e. 10.5 and 13.0 g kg^−1^, while rest of the groups showed an insignificant (*P ˃* 0.05) lower values of ash content in their body.

**Table 2:** Carcass composition of rainbow trout, *Oncorhynchus mykiss* fingerlings fed diets containing varying levels of dietary valine for 8-weeks (mean values of 3 replicates + SEM; *n* = 3)^*^.

	Dietary val levels (g kg^−1^)							
	Initial	10.5	13.0	15.5	18.0	20.5	23.0	P-value
Moisture (%)	78.51 ± 0.47	77.76 ± 0.28^a^	76.50 ± 0.22^b^	74.49 ± 0.23^c^	72.80 ± 0.18^d^	72.56 ± 0.19^d^	72.10 ± 0.24^e^	0.00107
Protein (%)	13.51 ± 0.15	12.16 ± 0.08^f^	13.42 ± 0.10^e^	15.22 ± 0.12^d^	17.45 ± 0.14^a^	16.64 ± 0.70^b^	16.02 ± 0.80^c^	0.01346
Fat (%)	3.52 ± 0.06	5.34 ± 0.07^f^	5.84 ± 0.04^e^	6.13 ± 0.07^d^	6.56 ± 0.06^c^	7.25 ± 0.09^b^	7.65 ± 0.08^a^	0.00214
Ash (%)	2.66 ± 0.05	3.27 ± 0.08^a^	2.76 ± 0.06^b^	2.34 ± 0.06^c^	2.10 ± 0.05^c^	2.24 ± 0.05^c^	2.30 ± 0.04^c^	0.01388

### Hematological parameters

In the present study, dietary Val levels affected hematological parameters of fingerling *O. mykiss* as indicated in Table 3. Hemoglobin (Hb), hematocrit (Hct) and red blood cell (RBC) count significantly (*P* < 0.05) improved with increasing concentrations of dietary Val up to 18.0 g kg^−1^ diet, thereafter significant decrease in these three parameters were seen. Likewise, significant (*P* < 0.05) differences in total leukocyte (WBC) counts were also observed in all the Val fed groups with highest WBC (3.15 × 10^4^ mm^−3^) count was occurred at lowest Val fed group i.e. 10.5 g kg^−1^. Erythrocyte sedimentation (ESR) rate was found lowest (1.67 mm h^−1^) at fish fed 20.5 g kg^−1^ Val diet, while highest ESR content (3.08 and 2.68 mm h^−1^) was registered in those groups that fed lower dietary Val quantities i.e. 10.5 and 13.0 g kg^−1^, respectively. Significant (*P* < 0.05) differences in mean corpuscular value (MCV) was also recorded with highest (175.06 fl) MCV was noted with fish fed higher Val level, while lowest MCV value was found for fish fed least Val containing diet i.e. 10.5 g kg^−1^. However, no significant (*P* > 0.05) differences in MCHC and MCH values were seen among all the treatments.

**Table 3:** Haematological indices of rainbow trout, *Oncorhynchus mykiss* fingerlings fed diets containing varying levels of dietary valine for 8-weeks (mean values of 3 replicates + SEM; *n* = 3)^*^

	Dietary val levels (g kg^−1^)							
	Initial	10.5	13.0	15.5	18.0	20.5	23.0	P-value
Hb (g dL^−1^)^a^	5.05 ± 0.06	6.78 ± 0.04^d^	8.58 ± 0.06^c^	9.89 ± 0.07^b^	10.96 ± 0.12^c^	9.54 ± 0.11^b^	9.08 ± 0.13^bc^	0.01154
Hct (%)^b^	20.32 ± 0.13	22.40 ± 0.12^d^	27.10 ± 0.12^c^	31.16 ± 0.08^b^	35.12 ± 0.19^a^	32.09 ± 0.14^b^	23.25 ± 0.08^d^	0.00318
RBC (mm h^−1^)^c^	1.45 ± 0.03	1.58 ± 0.06^cd^	1.89 ± 0.06^c^	2.18 ± 0.07^b^	2.63 ± 0.06^a^	2.36 ± 0.04^b^	2.13 ± 0.06^b^	0.01418
WBC (× 10^3^/mm^3^)^d^	2.49 ± 0.12	3.15 ± 0.05^a^	2.70 ± 0.04^b^	2.43 ± 0.05^d^	2.24 ± 0.08^e^	2.39 ± 0.07^d^	2.57 ± 0.09^c^	0.01444
ESR (mm h^−1^)^e^	2.36 ± 0.14	3.08 ± 0.10^a^	2.68 ± 0.09^b^	1.95 ± 0.12^c^	1.87 ± 0.06^c^	1.67 ± 0.05^d^	1.71 ± 0.12^d^	0.01038
MCV (fl)^f^	161.13 ± 1.30	157.10 ± 1.42^d^	166.26 ± 1.19^b^	162.78 ± 1.43^c^	164.16 ± 1.42^b^	167.20 ± 1.55^b^	175.06 ± 1.13^a^	0.16362
MCH (pg)^g^	41.50 ± 0.31	40.66 ± 0.54^a^	37.18 ± 0.58^b^	38.55 ± 0.27^b^	37.35 ± 0.33^b^	36.67 ± 0.48^b^	37.07 ± 0.22^b^	0.11748
MCHC (g dL^−1^)^h^	25.52 ± 0.39	26.29 ± 0.28^a^	23.96 ± 0.22^b^	24.02 ± 0.25^b^	24.45 ± 0.36^b^	24.69 ± 0.22^b^	24.61 ± 0.34^a^	0.11992

^a^Hb, Hemaglobin; ^b^Hct, Hematocrit; ^c^RBC, Red blood cell; ^d^WBC, White blood cell; ^e^ESR, Erythrocyte sedimentation rate; ^f^MCV, Mean cell volume; ^g^MCH, Mean corpuscular hemoglobin; ^h^MCHC, Mean corpuscular hemoglobin concentration.

### Plasma indices

**Figure 2:**
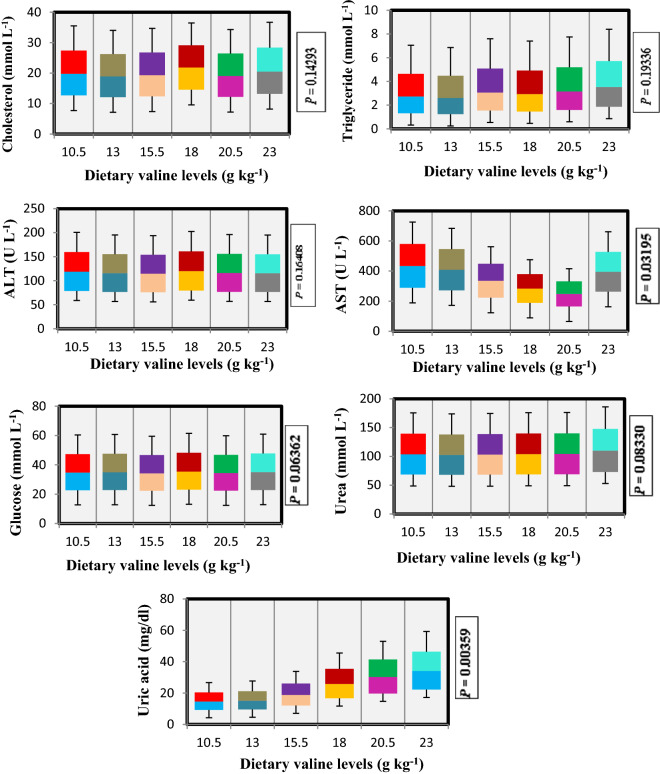
Blood plasma parameters of rainbow trout, *Oncorhynchus mykiss* fingerlings fed diets containing graded levels of valine (g kg^−1^).

The plasma indices of rainbow trout are shown in Fig. 2, which indicated no significant (*P* > 0.05) differences in plasma cholesterol (CHO), triglycerides (TG), alanine transaminase (ALT), glucose and urea contents. While, significant reduction in aspartate transaminase (AST) was seen with increasing Val diet up to 18.0 g kg^−1^ and afterwards an increase in AST content trend was noted. However, uric acid content increased constantly with increasing Val levels and reached its maximum value at 23.0 g kg^−1^ Val fed diet.

### Non-specific immune response

**Figure 3:**
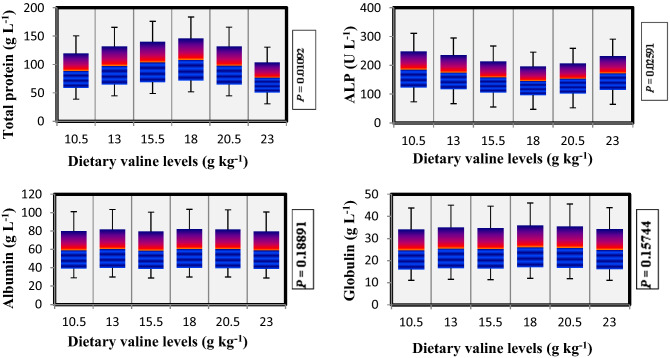
Non-specific immune response of rainbow trout, *Oncorhynchus mykiss* fingerlings fed diets containing graded levels of valine (g kg^−1^).

During the present study, effect of Val on non-specific immune response parameters was also analyzed and the findings are presented in Fig. 3. Plasma total protein showed significantly (*P* < 0.05) elevated trend with increasing dietary Val level up to 18.0 g kg^−1^ diet, thereafter decrease in plasma total protein was noted, whereas as alkaline phosphatase (ALP) content showed a decreasing trend with each incremental Val concentrations. Contrary to this, the albumin and globulin contents showed no significant (*P* > 0.05) differences among all the treatments.

### Intestinal enzyme activities

**Figure 4:**
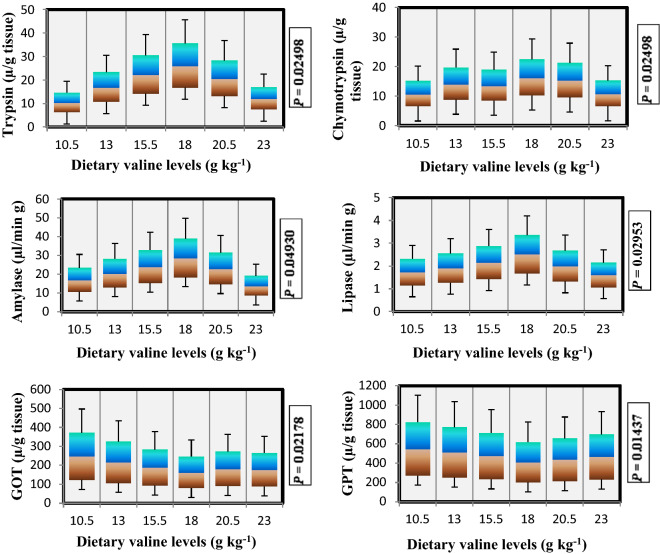
Digestive intestinal enzyme activities of rainbow trout, *Oncorhynchus mykiss* fingerlings fed diets containing graded levels of valine (g kg^−1^).

The results of digestive intestinal enzyme activities of rainbow trout fed varied levels of Val are presented in Fig. 4. Significant (*P* < 0.05) differences were noted in trypsin, chymotrypsin, amylase and lipase activities with increasing concentrations of dietary Val up to 18.0 g kg^−1^ Val fed diet, where higher activities of these constituents were seen which indicated that at this particular Val level fish utilize this amino acid more efficiently for growth and other activities including enzymatic activities. While as glutamate oxaloacetate transaminase (GOT) and glutamate pyruvate transaminase (GPT) activities decreased with the increase of dietary Val levels up to 18.0 g kg^−1^, afterwards decrease in these parameters were noted.

### Intestinal antioxidant status

Intestinal antioxidant variables were also carried out and the observations are presented in Table 4. The contents of superoxide dismutase (SOD), catalase (CAT), glutathione peroxidase (GPx) and glutathione reductase (GR) showed increased pattern significantly (*P* ˂ 0.05) with each incremental levels of dietary Val up to 18.0 g kg^−1^ diet, and thereafter decline in these parameters were also noted. Contrary to this, the contents of malondialdehyde (MDA) significantly (*P ˂* 0.05) decreased at each incremental level of dietary Val with maximum value was reported in fish fed initial diet i.e. 10.5 g kg^−1^.

**Table 4:** Effects of dietary valine levels on intestinal antioxidant enzymes of rainbow trout, *Oncorhynchus mykiss* fingerlings for 8-weeks (mean values of 3 replicates + SD; *n* = 3).

	**Dietary val levels (g kg**^**−1**^**)**						
	**10.5**	**13.0**	**15.5**	**18.0**	**20.5**	**23.0**	**P-value**
SOD (µ mg^−1^ protein)^a^	45.9 ± 0.72^d^	47.10 ± 0.82^bc^	48.06 ± 0.43^ab^	49.13 ± 0.47^a^	37.34 ± 0.52^e^	26.12 ± 0.62^f^	0.04578
CAT (µ mg^−1^ protein)^b^	0.56 ± 0.41^e^	0.96 ± 0.92^d^	1.22 ± 0.82^c^	1.52 ± 0.72^a^	1.40 ± 0.58^b^	1.20 ± 0.64^c^	0.02313
GPx (µ mg^−1^ protein)^c^	20.25 ± 0.97^d^	24.16 ± 1.02^c^	25.95 ± 1.06^b^	27.76 ± 0.94^a^	25.03 ± 1.04^b^	21.66 ± 0.93^d^	0.03962
GR (µ g^−1^ protein)^d^	15.44 ± 0.01^d^	18.56 ± 0.04^bc^	20.62 ± 0.04^b^	22.80 ± 0.07^a^	19.70 ± 0.09^bc^	13.05 ± 0.10^e^	0.01786
MDA (µ g^−1^ protein)^e^	0.56 ± 0.22^a^	0.46 ± 0.15^b^	0.32 ± 0.09^c^	0.22 ± 0.05^d^	0.24 ± 0.12^d^	0.30 ± 0.17^c^	0.01715

^a^SOD: Superoxide dismutase.

^b^CAT: Catalase.

^c^GPx: Glutathione peroxidase.

^d^GR: Glutathione reductase.

^e^MDA: Malondialdehyde.

### Relative gene expression of target of rapamycin (TOR) and 4E-BP in fish muscle

**Figure 5:**
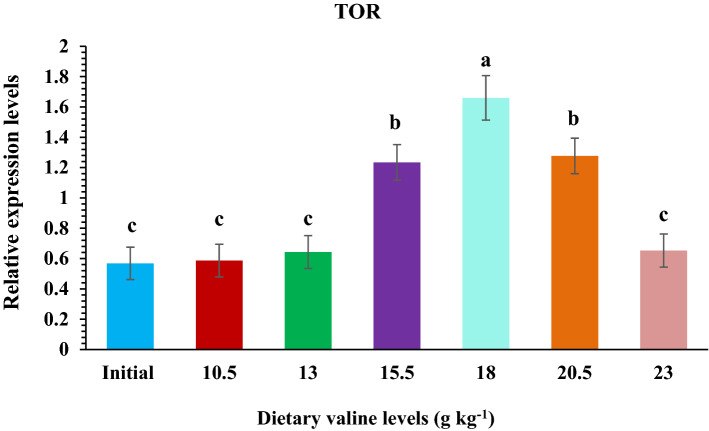
Relative expression level of target of rapamycin (TOR) gene in the muscle of rainbow trout, *Oncorhynchus mykiss* fingerlings fed varied dietary Val levels (g kg^−1^).

The effects of dietary Val on relative TOR and 4E-BP mRNA expression levels in rainbow trout are presented in Fig. 5,6. The relative expression of TOR and 4E-BP mRNA levels in muscle were observed highest in fish fed 18.0 g kg^−1^ Val diet compared to those fed remaining Val containing diets.

**Figure 6:**
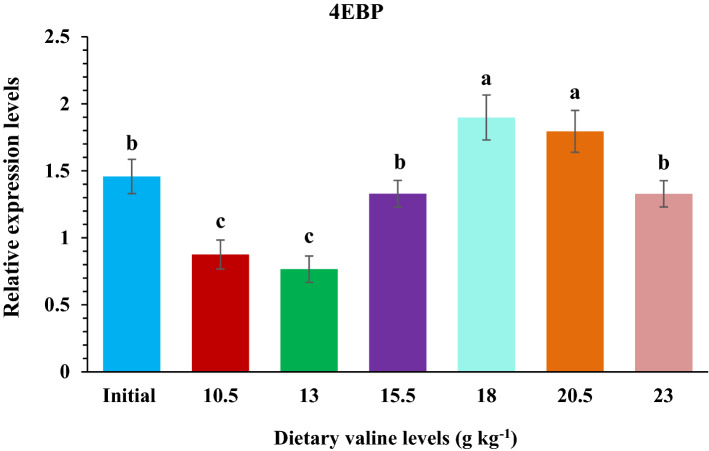
Relative expression level of elF-4E binding protein (4E-BP) gene in the muscle of rainbow trout, *Oncorhynchus mykiss* fingerlings fed varied dietary Val levels (g kg^−1^).

## Discussion

The branched chain amino acid Val along with leucine and isoleucine plays a significant role in protein synthesis and optimal growth of the fish. The main function of Val is to produce propionyl-CoA, the glycogenic precursor of succinyl-CoA^9^. It has been used as a supplement for fish growth along with leucine and isoleucine, besides also involved in the body to produce several biochemical compounds which mainly help in energy production^9^.The findings of the present study based on LWG%, FCR and PER indicated that the optimum dietary Val requirement was estimated to be 18.19 g kg^−1^ dry diet, corresponding to 40.42 g kg^−1^ of dietary protein, which is very close to the values reported on other fish species such as *Cirrhinus mrigala*^9^ 38.0 g kg^−1^, rainbow trout^13^ 38.5–41.0 g kg^−1^, Jian carp^23^ 40.0 g kg^−1^ and blunt snout bream 38.8 g kg^−1^, but higher than the values reported for red sea bream^1^ 20.0 g kg^−1^, *Catla catla*^56^ 30.9 g kg^−1^, red drum^21^ 32.0–35.0 g kg^−1^ and juvenile hybrid grouper^17^ 31.6 g kg^−1^. However, the Val requirement on trout in the present study is lower than that reported for grass carp^26^ 47.7–48.2 g kg^−1^ and golden pompano^25^ 46.0–47.0 g kg^−1^. These data revealed substantial disparity in the optimal Val requirement among fish species.

The huge variations recorded in the dietary Val requirement among various fish species could be attributed to various factors such as experimental designs, intact source of protein^57^, feed formulation, composition of test diets and utilization of different mathematical approaches^9,16,34,58,59^. Besides these above factors, various other attributes are also responsible for variations in optimum requirement of fish like water temperature, dissolved oxygen, source of food, size, age of the fish, assimilation rate, amino acids form and the energy utilized from feed-stuff and stocking density^60–62^. Moreover, several other physiological and metabolic needs for particular amino acid could also differ among different species, which may lead to differences in essential amino acid requirements^63,64^. Bureau et al.^65^ suggested that different environmental factors can cause a significant impact on nutritional requirement of fish.

In the present study, the lower LWG% observed in fish fed 10.5 or 23.0 g kg^−1^ dietary Val levels compared to that fed 18.0 g kg^−1^ dietary Val indicated that either deficient or excessive dietary Val levels could induce a decrease in growth performance and feed utilization. Similar observations were also recorded for *C. mrigala*^9^; *C. catla*^66^; *Oreochromis niloticus*^24^; *Epinephelus fuscoguttatus* x *E. lanceolatus*^17^. The reduction in growth performance due to above facts might be attributed to imbalance of amino acids, the loss of appetite caused by Val and poor feed utilization efficiency^9,23^. The another reason that could affect growth performance of fish is the presence of excess amount of specific amino acid, that is being consumed by the fish, which resulted demand of extra energy for the deamination or excess accumulation of extra nitrogen in the body as well as oxidation of ketones and harmful metabolites^66–68^. For *C. mrigala*, Ahmed and Khan^9^ reported that the reduced growth and feed efficiency induced by an excess of one of the BCAAs was due to the extra energy expenditure directed towards the deamination and excretion of the BCAAs in excess. In the similar manner, some authors reported that the reduction in growth of fish fed excess valine may be due to the antagonistic effects between valine and other BCAAs in different fish species including rainbow trout^69,70^.

The whole-body composition of fish is always treated as an indicator of fish quality. Several internal and external markers like fish size, rearing conditions, strain, season including growth, feeding rate and nature of the diet are well recognized to influence the fish body composition^71–81^. The whole-body composition of rainbow trout fed different concentrations of dietary Val in this study varied substantially. Whole body moisture content decreased significantly with the elevation of dietary Val concentrations and was maximum at higher level, while whole body fat content ameliorated with increasing Val concentrations with maximum fat content was noted at highest Val fed diet. Previous studies about the whole body composition of fish revealed that body fat and moisture contents are inversely related to each other^82,83^. Similar results have also been reported for Indian major carp, *C. mrigala*^8^. Whole body protein also elevated with increasing Val concentrations up to 18.0 g kg^−1^ Val fed diet and thereafter decreased in protein content was noted. Our result on fluctuation in body protein with respect to the elevation of each testing amino acid is in accordance with the finding on other fish species such as *C. mrigala*^9^; *L. rohita*^66^ and *C. catla*^84^. This rise in whole body protein content could be because of interest of several proteins or due to amino acid transamination responses occurring in fish body^9^. Body ash content of rainbow trout did not showed any difference with respect to the elevation of Val in the diet and remained constant among all the doses, except at lower Val fed diets i.e. 10.5 and 13.0 g kg^−1^. Higher ash contents reported at lowest Val level diets may be due to lower muscle deposition, which proportionally increases organic and inorganic content in the fish body^85^. Moreover, HSI is an important indicator applied to studies on nutritional requirements^56,86–88^, as it indicates the nutritional and physiological status of fish, besides provide an information pertaining to energy reserves in the body^89^. In the present research work, the lowest dietary Val concentrations resulted in higher HSI values in fish compared to the fish fed high levels of dietary Val, which could resultant in higher hepatic activity due to Val deficiency that resulted in the deamination of unused amino acid or their transamination into intermediate metabolic products for use as an energy source^84^. Increased HSI content in Val and isoleucine deficient diets have previously been documented in *C. catla*^84^ and *Channa punctatus*^68^, respectively.

Hematological variables are treated as an important markers in response to dietary manipulations^28,90–92^. These variables in fish can also be a critical indicator to assess alterations in circulatory system by toxic substances^93^. In the current research work, hematological parameters of rainbow trout fed with varied levels of Val showed significant differences. Among them, hematocrit assay is a reliable index in the aquaculture and fishery management for checking the anemic condition as well as fish health relative to nutrition, disease and stress status^94^. Highest Hb, Hct and RBC values were noted in the fish fed 18.0 g kg^−1^ Val diet, thereafter reduction in these three variables were recorded. The higher values for these variables at optimal dietary Val level might be ascribed to expansion in fish growth gave a capable degree of blood oxygen transport framework from respiratory systems to tissues^95^, as well as due to higher metabolic demand. A diminished trend in ESR was noted with increasing concentrations of dietary Val and was least in the fish fed 20.5 g kg^−1^ Val diet, which could be attributed to high blood consistency, resultant an elevation in erythrocytes count. Erythrocyte indices (MCV, MCH and MCHC) have a particular importance in the diagnosis of anemia in most animals including fish^96^. In the present study, fish fed lower and higher dietary Val containing diets i.e. 10.5 and 23.0 g kg^−1^ actuated macrocytic anemia, because higher values were noted in these levels. Neverthless, no significant differences in MCH and MCHC data were acquired, which is accordance with our previous findings on another amino acids i.e. leucine^55^ and isoleucine (unpublished).

The blood plasma variables are generally treated as health condition indicators of fish and in response to nutritional supplements^33^. In the present study, plasma CHO, TG, ALT, glucose and urea contents were not influenced with increased Val concentrations, which is in agreement with the findings for other fish species for example, red sea bream^1^; blunt snout bream^22^ and Nile tilapia^24^. In the study, AST parameter demonstrated a decreased pattern with augmentation of dietary Val up to 18.0 g kg^−1^ diet indicating an enhancement in fish health status, which is in agreement with the study for *O. niloticus*^24^. Moreover, uric acid contents in the current research work constantly increased with increasing Val concentration and reached maximum at 23.0 g kg^−1^ Val diet.

In addition, branched-chain amino acids are associated in different immune functions in fish and also up-regulate critical metabolic pathways in response to infectious pathogens^69,97,98^. The total protein concentration in plasma has been used as a health indicator, stress marker and to check nutritional condition in fish^99^. In the present study, significant increase in plasma total protein level was noted by increasing Val levels up to the fish fed 18.0 g kg^−1^ Val diet. Xiao et al.^24^ also suggested that appropriate supplementation of Val can positively influence the non-specific immune reaction of juvenile Nile tilapia. Similar perceptions were reported for juvenile hybrid grouper and red sea bream fed with varied concentrations of Val diet^1,17^. While ALP as a regulative enzyme is associated with many vital functions and take part in the process of nutrients like fat, glucose, calcium and inorganic phosphatase absorption^100^. We noticed decreased ALP pattern with fish fed elevated Val in the diets, which supports the finding of Dong et al.^23^ for juvenile Jian carp. However, in the present research work, we did not found any significant differences in plasma albumin and globulin contents of the fish fed varied concentrations of Val.

Fish have a high adaptability in their digestive processes, where intestine is treated as the main site at which fish easily digest and absorb nutrients, and its structural integrity plays a vital role in the maintenance of effective digestion and absorption^16,101^. Several studies pertaining to the activities of digestive enzymes in fish showed that enzymatic activity is influenced by the diet ingested or by feeding habits^102–104^. In the present study, there were significant improvement in trypsin chymotrypsin, amylase and lipase activities up to the fish fed 18.0 g kg^−1^ Val diet, showing that suitable dietary Val could improve digestion capacity in fish. Similar perceptions were accounted for juvenile jian carp, *C. carpio* in hepatopancreas and intestine^23,35^. Moreover, GOT and GPT are mainly liver enzymes, but they are also found in several organs or tissues. In the present study, the activities of GOT and GPT were decreased with increasing concentrations of dietary Val up to a certain point, thereafter reduction in these parameters were observed. To the best of our knowledge, no work has so far been carried out on intestinal GOT and GPT activities when fed with varied levels of dietary Val, however, some studies pertaining to these activities in hepatopancreas and muscle have been established on other fish species, where they observe significant significant increment in these parameters in hepatopancrease and muscle of *Cyprinus carpio* fed threonine and tryptophan diet, respectively^51,52^.

The intestinal epithelial integrity in fish plays a crucial role in maintaining the normal function of physical barriers, but intestinal epithelial cells are very sensitive to oxidative damage^105^. To fight oxidative damage, fish possess several antioxidant enzymes, which includes SOD), CAT, GPx, GR and MDA^106^. Among antioxidant enzymes, the activity of SOD indirectly reflected the ability of removing the oxygen free radicals in the fish body by regulating superoxide radical’s dismutation to hydrogen peroxide, which can be eliminated by GPx^41^. SOD can efficiently eject the reactive oxygen species (ROS) in the fish body, results in generating hydrogen peroxide, which may in turn be detached by the activities of CAT. SOD and CAT also take part in several reactions independently^24^. In the present research work, SOD, CAT, GPx and GR activities were positively influenced with each incremental dietary Val levels up to 18.0 g kg^−1^, which indicated an enhancement in the ability of scavenging free radical in the intestine of rainbow trout at optimum dietary Val concentration. Similar results were also noted in other fish species such as juvenile hybrid grouper, *Epinephelus fuscoguttatus* ♀ × *Epinephelus lanceolatus* ♂^17^, golden pompano, *T. ovatus*^25^ and Nile tilapia, *O. niloticus*^24^. MDA is treated as an important biomarker for protein oxidation and lipid peroxidation^107^, and it has been reported that MDA levels indirectly reflected the severity of fish body cell from free radicals attack^41^. Our findings found a significant reduction in MDA content up to 18.0 g kg^−1^, which demonstrated that appropriate dietary Val concentration could restrain the lipid per-oxidation of rainbow trout. Overall, the above data indicated that appropriate supplementation of Val levels could improve non-specific immune response variables of rainbow trout fingerling.

In fish, the activation of TOR signaling cascade by dietary amino acids is reported, thereby promoting protein synthesis^108–110^. Besides acting as a substrate for protein synthesis, Val has also been considered as a key regulator of several biological and physiological processes in fish. For instance, Val plays a crucial role in regulating the TOR signaling pathway in response to nutritional status, resultant to protein synthesis and growth of fish^17^. In the TOR signaling pathway two downstream effectors, 4E-BP and S6K1, are the most important rate determining factors in protein synthesis^111,112^. Many amino acids, including tryptophan^52^, leucine^22,113,114^ and arginine^115–117^, have revealed to up-regulate fish growth performance and promote protein synthesis through the TOR signaling pathway. However, there are only few studies reported on the effect of Val on fish protein synthesis and its inter-relationship with TOR signaling pathway. There the present study aimed to explore the influence of dietary Val on TOR signaling pathway, which showed that relative expression of TOR gene was positively progressed by dietary Val concentrations up to 18.0 g kg^−1^ dry diet, and thereafter a reduction pattern was noted. Similar trends have been previously observed in our laboratory on rainbow trout fed with graded dietary leucine^55^ and isoleucine levels (under publication), and also in other fish species such as jian carp^29^, blunt snout bream^57^ and hybrid grouper^44^. Wacyk et al.^118^ also reported decreased trend in rainbow trout fed deficient branched chain amino acids diet. However, in this study the fish fed 13.0 and 15.5 g kg^−1^ Val containing diets had significantly lower relative 4E-BP mRNA expression levels compared to other groups and both TOR and 4E-BP levels were highest at 18.0 g kg^−1^ Val diet. These resulted is in supportive with the study in hybrid grouper^44^ fed varied levels of Val diet. The current results suggest that Val probably acts on the growth performance of fish by modulating the transcriptional regulation of its downstream effectors 4E-BP and S6K1. However, whether Val affects phosphorylation of the target proteins in TOR signaling pathway needs further exploration.

Our findings suggest that diets containing 18.19 g kg^−1^ Val in the diet would be useful for the improvement of growth performance, hemato-biochemical parameters and enhanced non-specific immune response, antioxidant status and digestive enzymatic activities in rainbow trout fingerlings. Besides, it has also been observed that optimal dietary Val also up-regulate an expression of TOR signaling pathway through its downstream effector i.e. 4E-BP, which can promote protein synthesis. Based on the quadratic regression analysis for growth parameters, the requirement of Val for rainbow trout was estimated to be 18.19 g kg^−1^ dietary Val, corresponding to 40.42 g kg^−1^ of dietary protein. Data from this study would be helpful in developing nutritionally sound complete diets for the intensive culture of rainbow trout.

## Materials and methods

### Experimental diets

Six isonitrogenous (450 g kg^−1^) and isoenergetic (20.90 kJ 100 g^−1^, gross energy (GE)) diets having a elevated content of Val 10.5, 13.0, 15.5, 18.0, 20.5, and 23.0 g kg^−1^ were supplied in six diet containing casein (fat-free), gelatin and L-crystalline amino acid premix (Table 5). The level of protein content in all the diets was fixed at 450 g kg^−1^, which was estimated optimum for maximum growth in our previous study^45^. L-crystalline amino acids in all the diets were used to adjust the amino acid content of the diets similar to that of 40% whole egg protein, excluding Val. The quantity of L-Val in all the diet was increased with the increment of 2.5 g kg^−1^ diet. The ratio of casein and gelatin was selected in all the diets on the basis of criteria that provide the lowest quantity of Val. In each incremental level, Val was ameliorated with the replacement of non-essential amino acids i.e. aspartic acid, serine and glycine, so as to maintain the uniform nitrogen level in all the diets. The doses of Val in all the diets were designed on the basis of previous studies on Val requirement of fish^119^. The procedure for preparation of experimental diets in this feeding experiment was same as adopted in earlier experiment^80^. The premixes of vitamin and mineral used in the study were prepared as per Halver^120^. Feed pellets were made with the help of pelletizer connected with a 2 mm die and were then dried in oven at 40 °C in order to decrease the moisture less than 100 g kg^−1^. Finally, the dry feed pellets were crushed, sealed in air tight bags at 4 °C until used.

**Table 5:** Composition of experimental diets used for estimating the dietary valine requirement of fingerlings rainbow trout, *Oncorhynchus mykiss.*

Experimental diets						
Ingredients (g kg^−1^, dry diet)	10.50	13.0	15.50	18.0	20.50	23.0
Casein^a^	140.0	140.0	140.0	140.0	140.0	140.0
Gelatin^b^	35.0	35.0	35.0	35.0	35.0	35.0
Amino acid mix^c^	336.29	337.09	337.59	338.09	338.59	339.09
Dextrin	197.90	196.70	195.90	195.10	194.40	193.60
Corn oil	50.0	50.0	50.0	50.0	50.0	50.0
Cod liver oil	100	100	100	100	100	100
Mineral mix^d^	40.0	40.0	40.0	40.0	40.0	40.0
Vitamin mix^d,e^	30.0	30.0	30.0	30.0	30.0	30.0
Carboxymethyl cellulose	60.0	60.0	60.0	60.0	60.0	60.0
Alpha cellulose	10.81	11.21	11.51	11.81	12.01	12.31
Total	1000	1000	1000	1000	1000	1000
Total valine	10.50	13.0	15.50	18.0	20.50	23.0
Calculated crude protein (g kg^−1^)	450.0	450.0	450.0	450.0	450.0	450.0
Analysed crude protein (g kg^−1^)	449.16	450.80	449.70	450.89	449.56	448.90
Gross energy^f^ (kJ g^−1^, dry diet)	20.9	20.9	20.9	20.9	20.9	20.9

^a^Crude protein (80%),^b^Crude protein (93%), Loba Chemie, India; ^c^Essential amino acids (g kg^−1^) arginine 17.71, histidine 4.48, isoleucine 23.80, leucine 22.17, lysine 15.94, methionine 11.57, phenylalanine 17.57, threonine 11.72, tryptophan 4.88, valine Variable. Non-essential amino acids cystine 9.04, tyrosine 10.83, alanine 15.30, aspartic acid variable, proline 14.34, serine variable, glycine variable (Loba Chemie, India). ^d^Halver 2002 mineral (AlCl_3_. 6H_2_O, 150; ZnSO_4_. 7H_2_O, 3000; CuCl,100; MnSO_4_.4-6H_2_O, 800; KI,150; CoCl_2_.6H_2_O,1000 mg kg^−1^; plus USP # 2 Ca (H_2_PO_4_)_2_. H_2_O, 135.8; C_6_H_10_CaO_6_ 327.0; C_6_H_5_O_7_Fe.5H_2_O, 29.8; MgSO_4_.7H_2_O, 132.0; KH_2_PO_4_ (dibasic), 239.8; NaH_2_PO_4_.2H_2_O, 87.2; NaCl, 43.5 (g kg^−1^); ^e^vitamin mix (choline chloride 5000: thiamin HCL 50; riboflavin 200; pyridoxine HCL 50; nicotinic acid 750; calcium pentothenate 500; inositol 2000; biotin 5.0; folic acid 15; ascorbic acid 1000; menadione 40; alpha-tocopheryl acetate 400; cyanocobalamine 0.1 (g kg^−1^). ^f^Calculated on the basis of fuel values 23.10, 20.21, 24.27, 16.02 and 37.65 kJ g^−1^ for casein, gelatin, amino acids, dextrin, and fat, respectively.

### Experimental design and feeding trial

Fingerling rainbow trout of equal size and good health from the same lot were obtained from local government hatchery unit to experimental station at University of Kashmir. The fish were first mild treated with potassium permanganate (KMnO_4_) to rule out any contamination, and were stocked under continuous flow through system for a fortnight by feeding a combination of practical diet in pellet form. Required number of fish was taken from the lot and was further acclimated to synthetic diet for 2 weeks^120^. After the period of acclimatization, 360 fingerlings (average initial body weight: 1.66 ± 0.02 g/fish; average initial length: 5.25 ± 0.34 cm/fish) were then randomly distributed in 70 L circular tanks (water volume 60 L) connected with water flow through system (2–2.5 Lmin^−1^) at the rate of 20 fish per tank for each diets in triplicates. The diets were fed at the rate of 5% BW day^−1^ on dry to wet-weight basis to each group^80^. The experimental period was lengthening up to 8 weeks and diets were offered at 09:00 and 17:00 h. Initial and weekly body weights were recorded on a top loading balance (Sartorius AG Germany, CPA224S). Troughs were siphoned off to remove fecal matters before feeding on daily basis. Accumulation of the diet at the bottom of the trough was avoided. Uneaten food, if any was siphoned off immediately, dried in a hot air oven and reweighed to measure the amount of food consumed. On the day of weekly measurements, fish were not offered any feed. Troughs were scrubbed and disinfected thoroughly with water and KMnO_4_ solution on the day they were batch weighed. Mortality, if any, was recorded.

### Water quality analysis

During the entire feeding trial, various physico-chemical parameters were estimated on alternate day basis. The water samples were taken early in the morning before routine feeding. Water temperature (12.7–16.2 °C) was monitored by using a mercury thermometer, while other parameters like dissolved oxygen (7.1–8.2 mg L^−1^), free carbon dioxide (6.1–15.2 mg L^−1^), and total alkalinity (70–83 mg L^−1^) were analyzed by standard methods of APHA^121^. The pH (6.9–7.5) was recorded with the help of digital pH meter (pH ep-HI 98,107, USA).

### Chemical analysis

At the beginning of the feeding trial, 30 fish specimens were taken up for initial whole-body proximate composition. After the completion of 8-week feeding trial, final weight of each tank was recorded. Eight fish were taken from each replicate groups and three sub-samples of each replicate (n = 3 × 3) were analyzed for final whole-body composition. Whole-body proximate analysis of test diets, initial and final body composition of fish samples were determined as per AOAC^122^: for moisture determination oven drying method at 105 ± 1 °C was employed for 22 h, crude protein was carried out with the help of N-Kjeldhal × 6.25 Kjeltec 8400 (FOSS Denmark), crude lipid was estimated by solvent extraction method with petroleum ether B.P. 40–60 °C using soxlet extraction technique (FOSS Avanti automatic 2050, Sweden), while ash content was measured by oven incineration at 650 °C for 2–4 h (Muffle furnace YSPL-532, India).

### Hematological analysis and hepatosomatic index (HSI)

Three fish were randomly subside from each dietary treatment replicate (n = 3 × 3) for hematological parameters and hepatosomatic index. Blood samples for analysis were collected in heparinized (Na-heparinised) capillary tubes from the haemal arch after severing the caudal peduncle. Pooled sample of blood was taken from each treatment group and stored in heparin coated vaccutainer plastic vials for further analysis. All the hematological analysis was carried out within 2 h after each extraction.

### Hemoglobin (Hb)

Hemoglobin content of blood was estimated by adopting the Drabkin^123^ method. 20 μl of blood was mixed with 5 ml of Drabkin solution (Loba chemie, India) in a test tube and left to stand for at least 15 min. The colour was developed and absorbance of the sample was measured spectrophotometrically (GENESYS 10S UV–VIS) at 540 nm and compared to that of hemoglobin standard (Ranbaxy, India).

### Hematocrit (Hct%)

Hematocrit was determined on the basis of sedimentation of blood. Heparinised blood (50 μl) was taken in a micro-hematocrit capillary (Na-heparinised) and spun in a micro-hematocrit centrifuge (REMI RM-12C, India) at 12,000 rpm for 5 min in order to obtain hematocrit values. The hematocrit values were measured with the help of hematocrit reader and the Hct values were presented as percentage by adopting the method given by Del Rio-Zaragoza et al.^124^.

### Total red blood cell (RBC) and white blood cell (WBC) count

For red blood cell (RBC) count, a blood sample (20 μl) was taken with the help of micro pipette (Finpipette, Finland) and diluted with Natt-Herrick’s^125^ diluent (1:200). The diluted sample was placed in a Neubauer improved hemocytometer (Marienfeld-Superior, Lauda-Konigshofen, Germany) and then the blood cells were counted under light microscope (Magnus-MLM, India). White blood cell count (WBC) was done with the same technique as used during the RBC count.

### Erythrocyte sedimentation rate (ESR)

ESR was measured by using Wintrobe tube method^126^. The anticoagulated blood was filled in a Wintrobe tube up to the zero mark on top and kept undisturbed in vertical position in a rack. This allows the sedimentation of erythrocytes. After one hour level of fall of the column of sediment was noted as ESR and expressed in mm per hour.

### Erythocyte indices (MCH, MCHC and MCV)

The erythrocyte indices including MCH, MCHC and MCV were calculated using standard formulae^127^.

After blood collection, the liver was removed from the same sample and weighed subsequently nearby 0.1 mg (Sartorius AG Germany CPA224S) for HSI analysis through following formula:

HSI (%) = (Liver weight / Total body weight) × 100 (Rajaguru^128^).

### Plasma analysis and non-specific immune response

Before the start of the experiment (n = 10) and after the completion of feeding trial, pooled blood (n = 2 × 3) samples were collected. Clotted blood in eppendrof tubes was centrifuged for 10 min using a high-speed centrifuge (REMI-12C) 4100 × g for 10 min at 4 °C. The separated plasma was then analyzed for plasma parameters and non-specific immune responses such as ALP, ALT, AST, glucose, urea, uric acid, total protein, albumin, globulin, cholesterol and triglycerides by using veterinary biochemistry analyzer (Vetscan VS2 Abaxis, USA).

### Intestinal enzyme activities

After dissection, intestine samples (n = 2 × 3) were collected immediately and homogenized in a 10 volumes of saline solution and then centrifuged at 6000 × g for 20 min at 4 °C to obtain a supernatant and was then freezed at -20 °C for further examination. Trypsin and chymotrypsin activities were analyzed as per Hummel^129^, while for assaying the activities of amylase and lipase, the technique given by Furné et al.^130^ was employed. GOT and GPT activities were assayed by the method given by^131,132^ .

### Intestinal antioxidant status

The activities of SOD, CAT, GPx, content of GR and MDA were analyzed with the help of kits (Nanjing Jiancheng Bioengineering Institute, Nanjing, China) and were assayed as per the protocol described by Zhang et al.^133^.

### Expression of TOR and 4E-BP genes by Real-Time PCR

For establishing the appropriate Val level, TOR signaling pathway mRNA gene expression was analyzed by assessing through real-time^52^. The extraction of total RNA from fish muscle was carried out by Trizol method (Thermo Fisher Scientific, Darmstadt, Germany) as per manufacturer´s guidelines followed by the quantification and purification of total RNA by spectrophotometer (GENESYS 10S UV–VIS Thermo Scientific). Then, 2 µl total RNA was taken to synthesize complementary DNA (cDNA) by use of cDNA synthesis kit (iScript™cDNA Synthesis Kit Biorad, Hercules, CA). The identified genes (TOR, 4E-BP and β-actin) were synthesized by using specific primers (Table 6). PCR confirmation of identified genes was done by running 2% agarose gel. The real-time PCR was employed for above genes (LightCycler 480 Roche, USA). PCR amplication was carried out for these genes with the help of Chromo 4TM fluorescence detector (Bio-Rad, Hercules, CA, USA) under different thermo cycling conditions. Melting curve analysis was performed by running a gradient from 95 to 50 ^0^C in order to confirm the presence of single PCR products. The 2^−∆∆^Ct method was carried out to find out the TOR and 4E-BP signaling gene expression comparative to those for β-actin as per the method given by^134^.

**Table 6:** Real-time PCR primer sequences of rainbow trout.

Target gene	Primer sequence (5′-3′)	Amplicon length (bp)	Annealing temperature (°C)	GenBank accession number
TOR^a^	^c^F = 5′CAGCCACACACTTTTACAGACC-3′	22	60.25	NM 001,124,235.1
	^d^R = 5′–AATCTTGGTGAGGTACGGCTG-3′	21	59.82	
4E-BP^b^	F = 5′- GACCAGGCGGATGACCATAA-3′	20	59.35	NM 001,165,149.2
	R = 5′- GCAGGAACTTCCGGTCGTAG-3′	20	61.40	
*β-actin*	F = 5′- CCCAAACCCAGCTTCTCAGT-3′	20	59.35	XM 021,615,845.1
	R = 5′- ATCCGCTGTTTCACCGTTCC-3′	20	59.35	

^a^TOR = Target of rapamycin.

^b^4E-BP = ELF4E-binding protein.

^c^F = Forward.

^d^R = Reverse.

### Statistical analysis

The data obtained with respect to various Val fed diets in the form of LWG%, FCR, PER, SGR% and body composition parameters were analyzed by one-way analysis of variance (ANOVA)^135,136^. To predict the significant differences (*P ˂* 0.05) among the groups, polynomial contrast method i.e. cubic regression^137^ was applied to analyze the growth data. Second-degree polynomial regression (Y = a + bx + cx^2^) analysis was also employed in growth data to determine the appropriate breaking points in response to dietary Val levels^137^. Statistical analyses were made with the help of origin software (version 8.5.1; San Clemente, CA).

### Ethical statement

During the present research work, all applicable international, national, and/or institutional guidelines for the care and use of animals were followed. All the protocols used have been approved by the Institutional Animal Ethics Committee (IAEC) prescribed by committee for the purpose of control and supervision of experiments on animals (CPCSEA) under R. No. 801/GO/RE/S/2003/CPCSEA.

### ARRIVE guidelines

The present study has been carried out in accordance with ARRIVE guidelines.

## Data Availability

The data used in this is including within the manuscript.

## References

[1] Rahimnejad S, Lee KJ (2013). Dietary valine requirement of juvenile red sea bream *Pagrus major*. Aquaculture.

[2] Meijer AJ (2003). Amino acids as regulators and components of non-proteinogenic pathways. J. Nutr..

[3] Jobgen WS, Fried SK, Fu WJ, Meininger CJ, Wu G (2006). Regulatory role for the arginine-nitric oxide pathway in metabolism of energy substrates. J Nutr. Biochem..

[4] Li P, Mai K, Trushenski J, Wu G (2009). New developments in fish amino acid nutrition: towards functional and environmentally oriented aquafeeds. Amino Acids.

[5] Ahmed I, Khan MA (2004). Dietary arginine requirement of fingerling Indian major carp, *Cirrhinus mrigala* (Hamilton). Aquacult. Nutr..

[6] FAO Food and agriculture organization. The state of world fisheries and aquaculture. Contributing to food security and nutrition for all. Rome, Italy, pp. 223 (2018).

[7] Hua K (2019). The future of aquatic protein: implications for protein sources in aquaculture diets. One Earth.

[8] Twibell RG, Griffin ME, Martin B, Price J, Brown PB (2003). Predicting dietary essential amino acid requirements for hybrid striped bass. Aquacult. Nutr..

[9] Ahmed I, Khan MA (2006). Dietary branched-chain amino acid valine, isoleucine and leucine requirements of fingerling Indian major carp, *Cirrhinus mrigala* (Hamilton). Br. J. Nutr..

[10] Peres MH, Oliva-Teles A (2009). The optimum dietary essential amino acid profile for gilthead seabream (*Sparus aurata*) juveniles. Aquaculture.

[11] Gatlin III, D. M. Principles of Fish Nutrition. SRAC Publication No. 5003 July 2010.

[12] Sahoo PR (2017). Early breeding and seed production of Indian major carps: Attributes of the innovation from an adaptive trial. Current Agricult. Res. J..

[13] Bae JY, Park G, Yun H, Hung SSO, Bai SC (2012). The dietary valine requirement for rainbow trout, *Oncorhynchuss mykiss*, can be estimated by plasma free valine and ammonia concentrations after dorsal aorta cannulation. J. Appl. Anim. Res..

[14] Fernstrom JD (2005). amino acid assessment workshop: branched chain amino acids and brain function. J. Nutr..

[15] Brosnan JT, Brosnan ME (2006). Branched-chain amino acids: enzyme and substrate regulation. J. Nnutr..

[16] NRC, National Research Council. Nutrient Requirements of Fish and shrimp. National Academy Press, Washington, DC, 376 pp. (2011).

[17] Zhou, Z. *et al*. Dietary valine levels affect growth, protein utilization, immunity and antioxidant status in juvenile hybrid grouper (*Epinephelus fuscoguttatus* x *Epinephelus lanceolatus*). *Br. J. Nutr*. (2020). 10.1017/S0007114520002858 32713354

[18] Hughes, S. G., Rumsey, G. L. & Nesheim, M. C. Dietary requirements for essential branched-chain amino acids by Lake trout. *Trans. Am. Fish. Soc.* **112**, 812–817 (1983).

[19] Wilson RP, Poe WE, Robinson EH (1980). Leucine, isoleucine, valine and histidine requirements of fingerling channel catfish. J. Nutr..

[20] Castillo, S. & Gatlin, D. M. III. Dietary requirements for leucine, isoleucine and valine (branched‐chain amino acids) by juvenile red drum *Sciaenops ocellatus*. *Aquac. Nutr*. **24**, 1056–1065 (2018).

[21] Ren MC (2015). Dietary leucine level affects growth performance, whole body composition, plasma parameters and relative expression of TOR and TNF-ɑ in juvenile blunt snout bream *Megalobrama amblycephala*. Aquaculture.

[22] Dong, M. *et al*. Growth, body composition, intestinal enzyme activities and microflora of juvenile Jian carp (*Cyprinus carpio* var. Jian) fed graded levels of dietary valine. *Aquac. Nutr.* **19**, 1–14 (2013).

[23] Xiao W (2018). Dietary valine requirement of juvenile Nile tilapia *Oreochromis niloticus*. Aquac. Nutr..

[24] Huang Z (2017). Effect of dietary valine levels on the growth performance, feed utilization and immune function of juvenile golden pompano *Trachinotus ovatus*. Aquac. Nutr..

[25] Luo JB (2014). The impaired intestinal mucosal immune system by valine deficiency for young grass carp (*Ctenopharyngodon idella*) is associated with decreasing immune status and regulating tight junction proteins transcript abundance in the intestine. Fish Shellfish Immunol..

[26] Lemaire P (1991). Changes with different diets in plasma enzymes (GOT, GPT, LDH, ALP) and plasma lipids (cholesterol, triglycerides) of sea-bass (*Dicentrarchus labrax*). Aquaculture.

[27] Adhikari, S., Sarkar, B., Chatterjee, A., Mahapatra, C. T. & Ayyappan, S. Effects of cypermethrin and carbofuran on certain hematological parameters and prediction of their recovery in a freshwater teleost, *Labeo rohita* (Hamilton). *Ecotox. Environ. Safe*. 220–226 (2004). 10.1016/j.ecoenv.2003.12.003 15157576

[28] Kader MA, Koshio S, Ishikawa M, Yokoyama S, Bulbul M (2010). Supplemental effects of some crude ingredients in improving nutritive values of low fishmeal diets for red sea bream *Pagrus major*. Aquaculture.

[29] Javed M, Ahmad I, Ahmad A, Usmani N, Ahmad M (2016). Studies on the alterations in haemological indices, micronuclei induction and pathological marker enzyme activities in *Channa punctatus* (spotted snakehead) perciformes, channidae exposed to the thermal power plant effluent. Springer Plus.

[30] Ihut A (2018). The influence of season variation on hematological parameters and oxidative stress for rainbow trout (*Oncorhynchus mykiss*). Bull. UASVM. Anim. Sci. Biotech..

[31] Xiao W (2019). Effect of dietary phenylalanine level on growth performance, body composition, and biochemical parameters in plasma of juvenile hybrid tilapia, *Oreochromis niloticus* × *Oreochromis aureus*. J. World Aquacult Soc..

[32] Tan X (2016). Effects of dietary leucine on growth performance, feed utilization, non-specific immune responses and gut morphology of juvenile golden pompano *Trachinotus ovatus*. Aquaculture.

[33] Deng DF (2010). Dietary lysine requirement of juvenile Pacific threadfin (*Polydactylus sexfilis*). Aquaculture.

[34] Zhao, J. *et al*. Effects of dietary isoleucine on growth, the digestion and absorption capacity and gene expression in hepatopancreas and intestine of juvenile Jian carp (*Cyprinus carpio* var. Jian). *Aquaculture* **368–369**, 117–128 (2012).

[35] Adham I, Nayernia K, Enzel W (1997). Spermatozoa lacking acrosin protein show delayed fertilization. Mol. Reprod. Dev..

[36] Svobodova Z (2001). Leukocyte profiles of diploid and triploid tench *Tinca tinca* L.. Aquaculture.

[37] Garcia-Rodriguez, T., Ferrer, M., Carrillo, J. C. & Castroviejo, J. Metabolic responses of *Buteo buteo* to long-term fasting and refeeding. *Comp. Biochem. Physiol*. **87A**, 381–386 (1997).

[38] Zakes, Z., Demska-Zakes, K., Szczepkowski, M., Rozynski, M. & Ziomek, E. Impact of sex and diet on hematological and blood plasma biochemical profiles and liver histology of pikeperch (*Sander lucioperca* (L.)). *Arch. Pol. Fish*. **24**, 61–68 (2016).

[39] Dawood, M. A. O. Nutritional immunity of fish intestines: important insights for sustainable aquaculture. *Rev. Aquacult*. **13**, 642–663 (2021).

[40] Jiang WD (2016). Changes in integrity of the gill during histidine deficiency or excess due to depression of cellular anti-oxidative ability, induction of apoptosis, inflammation and impair of cell-cell tight junctions related to Nrf2, tor and Nf-κb signaling in fish. Fish Shellfish Immunol..

[41] Li B (2007). Isolation and identification of antioxidative peptides from porcine collagen hydrolysate by consecutive chromatography and electrospray ionization-mass spectrometry. Food Chem..

[42] Petro TM, Bhatacharjee JK (1981). Effect of dietary essential amino acid limitations upon the susceptibility to *Salmonella typhimurium* and the effect upon humoral and cellular immune responses in mice. Infect. Immun..

[43] Tsukishiro T (2000). Effect of branched-chain amino acids on the composition and cytolytic activity of liver-associated lymphocytes in rats. J. Gastroenterol. Hepatol..

[44] Nakamura, H. Plasma amino acid profiles are associated with insulin, C-peptide and adiponectin levels in type 2 diabetic patients. *Nutrition & Diabetes,* **4***,* e133 (2014). 10.1038/nutd.2014.32 PMC4183973 25177913

[45] Weichhart T, Saemann MD (2009). The multiple facets of mTOR in immunity. Trends Immunol..

[46] Mareco EA, Vieira VL, Power DM, Johnston IA (2016). Comparison of the transcriptional responses of skeletal muscle and bone to a flooding dose of leucine in the gilthead sea bream (*Sparus aurata*). Comp. Biochem. Phys. A..

[47] Lansard M (2009). Hepatic protein kinase B (Akt)-Target of Rapamycin (TOR)-Signalling pathways and intermediary metabolism in rainbow trout (*Oncorhynchus mykiss*) are not significantly affected by feeding plant based diets. Br. J. Nutr..

[48] Lansard M, Panserat S, Plagnes-Juan E, Seiliez I, Skiba-Cassy S (2010). Integration of insulin and amino acid signals that regulate hepatic metabolism-related gene expression in rainbow trout: role of TOR. Amino Acids.

[49] Seiliez I (2011). Dietary carbohydrate-to-protein ratio affects TOR signaling and metabolism related gene expression in the liver and muscle of rainbow trout after a single meal. Am. J. Physiol. Regul. Integr. Comp. Physiol..

[50] Feng, L. *et al*. Threonine affects intestinal function, protein synthesis and gene expression of TOR in Jian carp (*Cyprinus carpio* var. Jian). *PLoS ONE,* **8**, e69974 (2013). 10.1371/journal.pone.0069974 PMC3724917 23922879

[51] Studies in vivo and invitro (2013). Tang, L. *et al*. Effect of tryptophan on growth, intestinal enzyme activities and TOR gene expression in juvenile jian carp (*Cyprinus carpio* var. Jian). Aquaculture.

[52] Zhou, M. *et al*. Boosting mTOR-dependent autophagy via upstream TLR4-MyD88-MAPK signalling and downstream NF-kappaB pathway quenches intestinal inflammation and oxidative stress injury. *EBioMedicine* **35**, 345–360 (2018). 10.1016/j.ebiom.2018.08.035 PMC6161481 30170968

[53] Ahmed, I. & Ahmad, I. Effect of dietary protein levels on growth performance, hematological profile and biochemical composition of fingerlings rainbow trout, *Oncorhynchus mykiss* reared in Indian himalayan region. *Aquacult. Rep*. **16**, 100268 (2020).

[54] Ahmad I (2021). Effects of dietary leucine levels on growth performance, hemato-biochemical parameters, liver profile, intestinal enzyme activities and target of rapamycin signaling pathway related gene expression in rainbow trout Oncorhynchus mykiss fingerlings. Aquacult. Nutr..

[55] Zehra S, Khan MA (2014). Dietary valine requirement of fingerling *Catla catla*. J. Appl. Aquac..

[56] Forster I, Ogata HY (1998). Lysine requirement of juvenile Japanese flounder *Paralichthys olivaceus* and juvenile red sea bream *Pagrus major*. Aquaculture.

[57] Encarnacao P (2004). Diet digestible energy content affects lysine utilization, but not dietary lysine requirements of rainbow trout (*Oncorhynchus mykiss*) for maximum growth. Aquaculture.

[58] Zehra S, Khan MA (2015). Dietary leucine requirement of fingerling *Catla catla* (Hamilton) based on growth, feed conversion ratio, RNA/DNA ratio, leucine gain, blood indices and carcass composition. Aquac. Int..

[59] De Silva SS, Gunasekera RM, Gooley G (2000). Digestibility and amino acid availability of three protein-rich ingredient-incorporateddiets by Murray cod *Maccullochella peelii* and the Australian shortfin eel *Anguilla australis*. Aquac. Res..

[60] Liu FJ (2014). Quantitative dietary leucine requirement of juvenile Pacific white shrimp, *Litopenaeus vannamei* (Boone) reared in low-salinity water. Aquacult. Nutr..

[61] Simmons L (1999). Dietary methionine requirement of juvenile Arctic charr *Salvelinus alpinus*. Aquacult. Nutr..

[62] Wilson RP, Poe WE (1985). Relationship of whole body and egg essential amino acid patterns to amino acid requirement patterns in channel catfish (*Zctalurus punctatus*). Comp. Biochem. Phys. A..

[63] Khan, K. U. *et al*. Whole-body amino acid pattern of juvenile, preadult, and adult pacu, *Piaractus mesopotamicus*, with an estimation of its dietary essential amino acid requirements. *J. World Aquac. Soc*. (2019).

[64] Bureau, D. P., Kaushik, S. J. & Cho, C. Y. Bioenergetics. In: JE Halver, RW Hardy (eds) Fish Nutrition, 3rd edn, pp. 2– 61. Elsevier Science, USA. (2002).

[65] Khan MA, Abidi SF (2007). Dietary isoleucine requirement of fingerling Indian major carp, *Labeo rohita* (Hamilton). Aquac. Nutr..

[66] Ren MC (2017). Dietary isoleucine requirement of juvenile blunt snout bream *Megalobrama amblycephala*. Aquac. Nutr..

[67] Sharaf Y, Khan MA (2020). Effect of dietary isoleucine level on growth, protein retention efficiency, haematological parameter, lysozyme activity and serum antioxidant status of fingerling *Channa punctatus* (Bloch). Aquac. Nutr..

[68] Ahmad, I., Ahmed, I., Fatma, S. & Peres, H. Role of branched chain amino acids on growth, physiology and metabolism of different fish species: A review. *Aquacu. Nutr.* (2021).

[69] Yamamoto T, Shima T, Furuita H (2004). Antagonistic effects of branched-chain amino acids induced by excess protein-bound leucine in diets for rainbow trout (*Oncorhynchus mykiss*). Aquaculture.

[70] Cho CY, Slinger SJ, Bailey HS (1976). Influence of level and type of dietary protein, and of level of feeding on feed utilization by rainbow trout. J. Nutr..

[71] Reinitz G, Hitzel FN (1980). Formulation of practical diets for rainbow trout based on desired performance and body composition. Aquaculture.

[72] Reinitz G (1983). Relative effect of age, diet and feeding rate on the body composition of young rainbow trout (*Salmo gairdneri*). Aquaculture.

[73] Storebakken, T. & Austreng, E. Ration level for salmonids. I. Growth, survival, body composition and feed conversion in Atlantic salmon fry and fingerlings. *Aquaculture* **60**, 189–206 (1987).

[74] Parazo MM (1990). Effect of dietary protein and energy level on growth, protein utilization and carcass composition of rabbitfish Siganus guttatus. Aquaculture.

[75] Lovell RT (1992). Comparison of satiate feeding and restricted feeding in channel catfish with various concentrations of dietary proteins in production ponds. Aquaculture.

[76] Hassan, M. A. & Jafri, A. K. Optimum feeding rate, and energy and protein maintenance requirements of young *Clarias batrachus* (L.) a cultivable catfish species. *Aquac. Fish. Manag*. **25**, 427–438 (1994).

[77] Panda S, Mishra K, Samantaray K (1999). Effect of feeding rate on the growth performance of *Channa punctatus* (Bloch) fry, and protein and energy requirement for their maintenance and maximum growth. J. Aquac..

[78] Adebayo OT, Balogun AM, Fagbenro OA (2000). Effect of feeding rates on growth, body composition and economic performance of juvenile clariid catfish hybrid (♀ *Clarias gariepinus* × ♂ *Heterobranchus bidorsalis*). J. Aquacult. Trop..

[79] Khan MA, Ahmed I, Abidi SF (2004). Effect of ration size on growth, conversion efficiency and body composition of fingerling mrigal, *Cirrhinus mrigala* (Hamilton). Aquac. Nutr..

[80] Ahmed I (2007). Dietary amino acid L-threonine requirement of fingerling Indian catfish, *Heteropneustes fossilis* (Bloch) estimated by growth and biochemical parameters. Aquac. Int..

[81] Dumas A, France J, Bureau D (2010). Modelling growth and body composition in fish nutrition: where have we been and where are we going?. Aquac. Res..

[82] Ye, W., Han, D., Zhu, X., Yang, Y., Jin, J. & Xie, S. Comparative study on dietary protein requirements for juvenile and pre-adult gibel carp (*Carassius auratus gibelio* var. CAS III). *Aquac. Nutr*. **23**, 755–765 (2017).

[83] Zehra S, Khan MA (2013). Dietary isoleucine requirement of fingerling catla, *Catla catla* (Hamilton), based on growth, protein productive value, isoleucine retention efficiency and carcass composition. Aquac. Int..

[84] Pianesso D (2015). Determination of tryptophan requirements for juvenile silver catfish (*Rhamdia quelen*) and its effects on growth performance, plasma and hepatic metabolites and digestive enzymes activity. Anim. Feed Sci. Tech..

[85] Coloso, R. M., Murillo-Gurrea, D. P., Borlongan, I. G. & Catacutan, M. R. Tryptophan requirement of juvenile Asian sea bass *Lates calcarifer*. *J. Appl. Ichthyol*. **20**, 43-. 47 (2004).

[86] Ahmed I (2012). Dietary amino acid l-tryptophan requirement of fingerling Indian catfish, *Heteropneustes fossilis* (Bloch), estimated by growth and haemato-biochemical parameters. Fish Physiol. Biochem..

[87] Farhat & Khan, M. A. Effects of varying levels of dietary l-histidine on growth, feed conversion, protein gain, histidine retention, hematological and body composition in fingerling stinging catfish *Heteropneustes fossilis* (Bloch). *Aquaculture* **404–405**, 130–138 (2013).

[88] Balawi HFAB (2011). Toxicity bioassay of lead acetate and effects of its sublethal exposure on growth, haematological parameters and reproduction in *Clarias gariepinus*. Afr. J. Biotechnol..

[89] Maheswaran, R., Devapaul, A., Muralidharan, S., Velmurugan, B. & Ignacimuthu, S. Haematological studies of freshwater fish, *Clarias batrachus* (L.) exposed to mercuric chloride. *Int. J. Integ. Biol*. **2**, 49–54 (2008).

[90] Shah, S.L. & Altindag, A. Alterations in the immunological parameters of tech (Tincatinca L. 1758) after acute and chronic exposure to lethal and sub lethal treatments with mercury, cadmium and lead. *Turk. J. Vet. Anim. Sci*. **29**, 1163–1168 (2005).

[91] Ahmed I (2014). Dietary amino acid L-methionine requirement of fingerling Indian catfish, *Heteropneustes fossilis* (Bloch-1974) estimated by growth & haemato-biochemical parameters. Aauac. Res..

[92] Vinodhini, R. & Narayanan, M. The impact of toxic heavy metal on the hematological parameters in common carp (*Cyprinus carpio* L.). *Iranian J. Environ. Health Sci. Eng*. 23–8 (2009).

[93] Mozanzadeh MT (2015). Reference intervals for haematological and plasma biochemical parameters in sobaity sea bream juveniles (*Sparidentex hasta*, Valenciennes 1830). Comp. Clin. Pathol..

[94] Nespolo RF, Rosenmann M (2002). Intraspecific allometry of haematological parameters in *Basilichthys australis*. J. Fish. Biol..

[95] Coles EH (1986). Veterinary Clinical Pathology.

[96] Habte-Tsion HM (2015). Threonine influences the absorption capacity and brush-border enzyme gene expression in the intestine of juvenile blunt snout bream (*Megalobrama amblycephala*). Aquaculture.

[97] Feng L (2017). Gill structural integrity changes in fish deficient or excessive in dietary isoleucine: Towards the modulation of tight junction protein, inflammation, apoptosis and antioxidant defense via NF-kB, TOR and Nrf2 signalling pathways. Fish Shellfish Immunol..

[98] Riche, M. Analysis of refractometry for determining total plasma protein in hybrid striped bass (*Morone chrysops* × *M. saxatilis*) at various salinities. *Aquaculture* **264**, 279−284 (2007).

[99] Tengjaroenkul B, Smith BJ, Caceci T, Smith SA (2000). Distribution of intestinal enzyme activities along the intestinal tract of cultured Nile tilapia *Oreochromis niloticus* L. Aquaculture.

[100] Almeida, A. P. G. *et al*. Composition of gastrointestinal content, protease and lipase activities in summer and winter of four freshwater siluriforms (Teleostei: Actinopterygii) with two different feeding habits. *Zoologia* **35**, e13286 (2018).

[101] Drewe KE, Horn MH, Dickson KA, Gawlicka A (2004). Insectivore to frugivore: Ontogenetic changes in gut morphology and digestive enzyme activity in the characid fish *Brycon guatemalensis* from Costa Rican rainforest streams. J. Fish Biol..

[102] Langeland M, Lindberg JE, Lundh T (2013). Digestive enzyme activity in Eurasian perch (*Perca fluviatilis*) and Arctic charr (*Salvelinus alpinus*). J. Aquac. Res. Dev..

[103] Solovyev MM, Izvekova GI, Kashinskaya EN, Gisbert E (2017). Dependence of pH values in the digestive tract of freshwater fishes on some abiotic and biotic factors. Hydrobiologia.

[104] Minghetti M, Drieschner C, Bramaz N, Schug H, Schirmer K (2017). A fish intestinal epithelial barrier model established from the rainbow trout (*Oncorhynchus mykiss*) cell line, RTgutGC.. Cell Biol. Toxicol..

[105] Martinez-Alvarez RM, Morales AE, Sanz A (2005). Antioxidant defenses in fish: biotic and abiotic factors. Rev. Fish. Biol. Fish..

[106] Rana T (2010). Effect of ascorbic acid on blood oxidative stress in experimental chronic arsenicosis in rodents. Food Chem. Toxicol..

[107] Wu C (2017). The effects of dietary leucine on the growth performances, body composition, metabolic abilities and innate immune responses in black carp *Mylopharyngodon piceus*. Fish Shellfish Immunol..

[108] He Y (2019). dl-Methionine supplementation in a low-fishmeal diet affects the TOR/S6K pathway by stimulating ASCT2 amino acid transporter and insulin-like growth factor-I in the dorsal muscle of juvenile cobia (*Rachycentron canadum*). Br. J. Nutr..

[109] Habte-Tsion, H. M. A review on fish immuno-nutritional response to indispensable amino acids in relation to TOR, NF-κB and Nrf2 signaling pathways: Trends and prospects. *Comp. Biochem. Physiol. B. Biochem. Mol. Biol. ***241**, 110389 (2020). 10.1016/j.cbpb.2019.110389 31812790

[110] Holz MK, Ballif BA, Gygi SP, Blenis J (2005). mTOR and S6K1 mediate assembly of the translation preinitiation complex through dynamic protein interchange and ordered phosphorylation events. Cell.

[111] Rong H (2020). Effect of hydroxyproline supplementation on growth performance, body composition, amino acid profiles, blood-biochemistry and collagen synthesis of juvenile Chu's croaker (*Nibea coibor*). Aquac. Res..

[112] Zou, T. *et al*. Effects of dietary leucine levels on growth, tissue protein content and relative expression of genes related to protein synthesis in juvenile gibel carp (*Carassius auratus gibelio* var. CAS III). *Aquac. Res*. **49**, 2240–2248 (2018).

[113] Zhou Z (2019). Effects of dietary leucine levels on growth, feed utilization, neuro-endocrine growth axis and TOR-related signaling genes expression of juvenile hybrid grouper (*Epinephelus fuscoguttatus* ♀× *Epinephelus lanceolatus* ♂). Aquaculture.

[114] Chen, G. F. *et al*. Effect of dietary arginine on growth, intestinal enzyme activities and gene expression in muscle, hepatopancreas and intestine of juvenile Jian carp (*Cyprinus carpio* var. Jian). *Br. J. Nutr.* **108**, 195–207 (2012). 10.1017/S0007114511005459 22013925

[115] Liang H (2016). Dietary arginine affects growth performance, plasma amino acid contents and gene expressions of the TOR signaling pathway in juvenile blunt snout bream *Megalobrama amblycephala*. Aquaculture.

[116] Wu M (2018). Dietary arginine affects growth, gut morphology, oxidation resistance and immunity of hybrid grouper (*Epinephelus fuscoguttatus* ♀× *Epinephelus lanceolatus* ♂) juveniles. Br. J. Nutr..

[117] Wacyk J (2012). Dietary protein source significantly alters growth performance, plasma variables and hepatic gene expression in rainbow trout (*Oncorhynchus mykiss*) fed amino acid balanced diets. Aquaculture.

[118] NRC, National Research Council. Nutrient Requirement of Fish. National Academy Press, Washington DC, USA. (1993).

[119] Halver, J. E. The vitamins. Pages 61–141 in Halver J.E., Hardy R.W. editors. Fish Nutrition. 3rd edn. Academic Press, San Diego, CA. (2002).

[120] APHA. Standard methods for the examination of water and wastewater (18^th^ ed), APHA, Washington DC (1992) pp: 1268.

[121] AOAC. Official methods of analysis of the association of official analytical chemist (16th ed.), AOAC, Arlington, VA, USA (1995).

[122] Drabkin, D.L. Spectrometric studies. XIV. The crystallographic and optimal properties of the hemoglobin of man comparison with those of other species. *J. Biol. Chem.* **164,** 703–723 (1946). 21001166

[123] Del Rio-Zaragoza OB (2008). Thermal stress effect on tilapia *Oreochromis mossambicus* (Pisces: cichlidae) blood parameters. Mar. Freshw. Behav. Physiol..

[124] Natt MP, Herrick CA (1952). A new blood diluent for counting the erythrocytes and leucocytes of the chicken. Poult. Sci..

[125] Wedemeyer GA (1983). Some potentials and limits of the 1 eucorit test as a fish health assessment method. J. Fish Biol..

[126] Dacie JV, Lewis SM (1991). Practical haematology.

[127] Rajaguru, A. Biology of two co-occurring tongue fishes, *Cynoglossus arel* and *C. lida* (Pleuronectiformes: cynoglossidae), from Porto Nova, southeast coast of India. *Fish. Bull.* **90**, 328–367 (1992).

[128] Hummel BCW (1959). A modified spectrophotometric determination of chymotrypsin, trypsin, and thrombin. Can. J. Biochem..

[129] Furné M (2005). Digestive enzyme activities in Adriatic sturgeon *Acipenser naccarii* and rainbow trout *Oncorhynchus mykiss*, A comparative study. Aquaculture.

[130] Bergmeyer H. U. & Bernt E. Glutamate-pyruvatetransaminase: UV-assay, manual method, in Methods of Enzymatic Analysis (Bergmeyer H. U., ed), Vol. 2, pp. 752–758. Academic Press, New York. (1974a).

[131] Bergmeyer H. U. & Bernt E. Lactate dehydrogenase: UVassay with pyruvate and NADH, in Methods of Enzymatic Analysis (Bergmeyer H. U., ed), Vol. 2, pp. 574–579. Academic Press, New York. (1974b).

[132] Zhang, X.D., Zhu, Y.F., Cai, L.S., Wu, T.X., 2008. Effects of fasting on the meat quality and antioxidant defenses of market-size farmed large yellow croaker (*Pseudosciaena crocea*). *Aquaculture* **280**, 136–139 (1976).

[133] Livak KJ, Schmittgen TD (2001). Analysis of relative gene expression data using realtime quantitative PCR and the 2^-∆∆C^(T) method. Methods.

[134] Snedecor, G. W. & Cochran, W. G. Statistical Methods (6th ed), Iowa State University Press, Ames, Iowa. pp: 593 (1967).

[135] Sokal, R. R. & Rohlf, F. J. Biometry. W.H Freeman and Company, New York (1981) pp:859.

[136] Yossa R, Verdegem M (2015). Misuse of multiple comparison tests and underuse of contrast procedures in aquaculture publications. Aquaculture.

[137] Zeitoun IH, Ulrey DE, Magee WT, Gill JL, Bergen WG (1976). Quantifying nutrient requirements of fish. J. Fisheries Board Can..

